# The Proteasomal ATPases Use a Slow but Highly Processive Strategy to Unfold Proteins

**DOI:** 10.3389/fmolb.2017.00018

**Published:** 2017-04-04

**Authors:** Aaron Snoberger, Raymond T. Anderson, David M. Smith

**Affiliations:** Department of Biochemistry, West Virginia University School of MedicineMorgantown, WV, USA

**Keywords:** ATPase, proteasome, PAN, 26S, proteasomal ATPase, Rpt, AAA, AAA+

## Abstract

All domains of life have ATP-dependent compartmentalized proteases that sequester their peptidase sites on their interior. ATPase complexes will often associate with these compartmentalized proteases in order to unfold and inject substrates into the protease for degradation. Significant effort has been put into understanding how ATP hydrolysis is used to apply force to proteins and cause them to unfold. The unfolding kinetics of the bacterial ATPase, ClpX, have been shown to resemble a fast motor that traps unfolded intermediates as a strategy to unfold proteins. In the present study, we sought to determine if the proteasomal ATPases from eukaryotes and archaea exhibit similar unfolding kinetics. We found that the proteasomal ATPases appear to use a different kinetic strategy for protein unfolding, behaving as a slower but more processive and efficient translocation motor, particularly when encountering a folded domain. We expect that these dissimilarities are due to differences in the ATP binding/exchange cycle, the presence of a trans-arginine finger, or the presence of a threading ring (i.e., the OB domain), which may be used as a rigid platform to pull folded domains against. We speculate that these differences may have evolved due to the differing client pools these machines are expected to encounter.

## Introduction

Virtually every cellular process relies on properly regulated protein degradation. Bacteria, archaea, and eukaryotes all have systems for targeted protein degradation (e.g., the ClpP protease in bacteria and the 20S proteasome in archaea and eukaryotes). Both ClpP and the 20S proteasome are capable of degrading unfolded proteins, but since their peptidase sites are sequestered on their hollow interior with only small pores through which substrates can enter, these proteases are not able to degrade folded proteins by themselves because they are too bulky to enter these narrow translocation pores. In order to stimulate degradation of folded proteins, regulatory ATPase complexes associate with the proteolytic complex and use the chemical energy from ATP hydrolysis to unfold and inject the folded proteins into the proteases' central chamber for degradation. While much is understood about this process, we do not have a detailed molecular understanding of how these different ATP-dependent machines engage with and forcibly translocate substrates for selective protein degradation (Smith et al., 2006; Finley, 2009; Alexopoulos et al., 2012; Bar-Nun and Glickman, 2012; Tomko and Hochstrasser, 2013; Mack and Shorter, 2016).

To date some of the better characterized regulatory complexes for the 20S proteasome are the heterohexameric 19S regulatory particle in eukaryotes (which forms the 19S–20S, or “26S” complex) and the homohexameric 19S homolog in archaea, PAN (Proteasome Activating Nucleotidase). One of the most extensively studied ClpP regulators is ClpX. In general, the 19S, PAN, and ClpX utilize ATP to: (1) bind and open the gate of their respective protease (Grimaud et al., 1998; Smith et al., 2005; Liu et al., 2006; Alexopoulos et al., 2013), (2) recognize proper substrates (Thibault et al., 2006; Peth et al., 2010; Smith et al., 2011; Kim et al., 2015), and (3) unfold and inject them into their protease's degradation chamber (Ortega et al., 2000; Singh et al., 2000; Prakash et al., 2004; Zhang et al., 2009; Erales et al., 2012). All three of these regulators are members of the AAA+ superfamily (ATPases associated with diverse cellular activities), but only PAN and the 19S ATPases belong to the same AAA sub-clade, which also contain the SRH region (Lupas and Martin, 2002). Due to the complexities of generating ubiquitinated globular substrates that could be degraded by the purified 26S proteasome, far more functional studies have been done on PAN and ClpX, which only require the presence of a small unfolded region (i.e., ssrA) to trigger substrate degradation (Hoskins et al., 2002; Benaroudj et al., 2003). Although they serve similar functions, ClpX and the proteasomal ATPases may not exhibit similar mechanochemical translocation mechanisms, which would not be unexpected since they each belong to different sub-clades of the AAA+ family. Recent functional studies suggest that they may also have different ATP-hydrolysis characteristics. For example, evidence suggests that ClpX hydrolyzes ATP in a semi-stochastic fashion (Sauer and Baker, 2011), whereas the proteasomal ATPases appear to use an ordered, sequential cycle with a specific “ortho” binding pattern (binding to neighboring subunits) which is subject to expected equilibrium binding considerations (Smith et al., 2011; Kim et al., 2015). Additionally, function-critical allostery between subunits is mediated by the proteasomal ATPase's trans-arginine fingers (Kim et al., 2015), which is lacking in ClpX (Kim and Kim, 2003). These differences in the structure and hydrolysis patterns of ClpX and the proteasomal ATPases suggest they may use distinct mechanical strategies to unfold proteins.

**Figure 1:**
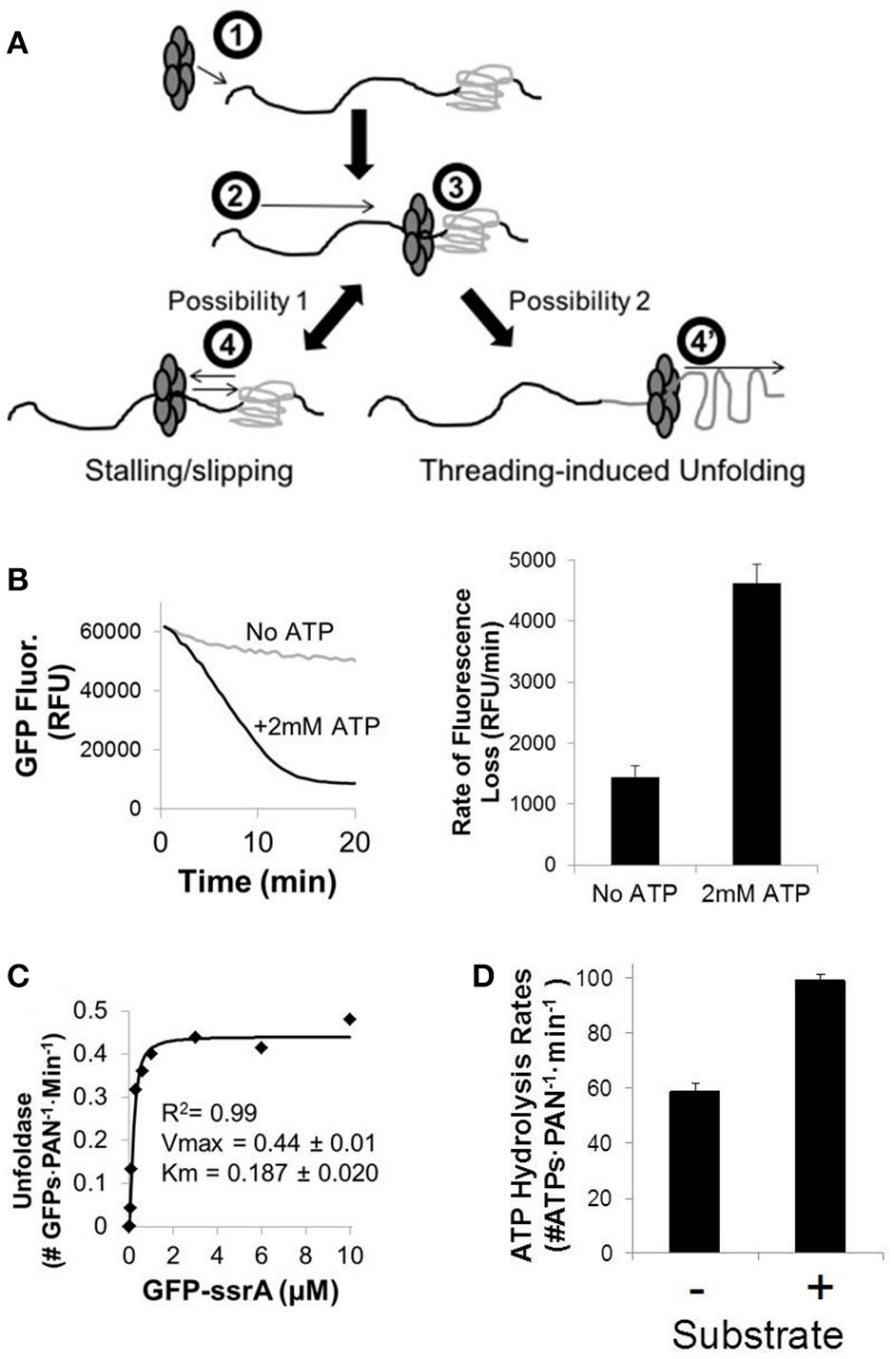
**Hexameric ATP-dependent proteases utilize energy from ATP hydrolysis to unfold substrates. (A)** Hexameric ATP-dependent proteases (e.g., ClpX or the proteasomal ATPases) (1) recognize their protein substrates and utilize energy from ATP hydrolysis to thread the protein through their central pore to (2) translocate along the unfolded region of the protein until they (3) reach a folded domain. (4) Less processive ATP-dependent proteases have a tendency to slip once they reach a more tightly folded domain, and if the ATP hydrolysis rates slow below a critical threshold they will stall and even slip backward before taking another run at the folded domain. (4′) More processive ATPases (or less processive ATPases after multiple runs at the folded domain) are able to drive through these more tightly folded domains to cause threading-induced unfolding of this protein domain, followed by further translocation along the protein. **(B)** The ATP-dependent GFPssrA substrate unfolding rate was measured in reaction buffer (see Materials and Methods Section) including 200 nM GFPssrA, 50 nM PAN, 400 nM T20S, and with and without saturating ATP (2 mM). Unfolding of GFPssrA was assessed by quantifying the steady-state rate of fluorescence loss (ex/em: 485/510). **(C)** GFPssrA unfolding kinetics were determined the same as in **(A)**, but with varying amounts of GFPssrA (from 0 to 10 μM). **(D)** Summary of ATPase rates with and without substrate for the proteasomal ATPases. ATPase rates for PAN were determined at 2 mM ATP using a kinetic NADH-coupled assay, with and without saturating GFPssrA (2 μM). Error bars are standard deviations from three independent experiments (*n* = 3).

Prior studies have shown that when ClpX is translocating on a protein and encounters a stably folded domain (e.g., GFP) it will often stop and even slip backward before taking another run at the folded domain. It's thought that this can occur over and over until spontaneous unfolding occurs after which ClpX quickly translocates onto the unfolded domain, trapping it, and preventing its refolding (Aubin-Tam et al., 2011; Maillard et al., 2011; Nager et al., 2011; Iosefson et al., 2015b; Rodriguez-Aliaga et al., 2016). ClpX may also perturb the folded domain prior to trapping. This likely continues until the whole domain is unfolded (Figure 1A). In this proposed model ClpX seems to function at high velocity, whereby quick trapping of unfolded intermediates (rather than brute force unfolding) is the primary strategy used to unfold the domain. Alternatively, one can think of this as a motor with high velocity, but with low processivity when it encounters an obstacle to translocation that causes slipping. Interestingly, the ATP hydrolysis rate of ClpX is ~100–500 ATPs per minute in the absence of substrate (Martin et al., 2005; Aubin-Tam et al., 2011; Maillard et al., 2011; Nager et al., 2011; Baytshtok et al., 2015; Iosefson et al., 2015a; Rodriguez-Aliaga et al., 2016), which is considerably faster than the ~30–60 ATPs per minute of the proteasomal ATPases (Hoffman and Rechsteiner, 1996; Kraut et al., 2012; Kim et al., 2015). Consistent with this high velocity, low processivity mechanism, ClpX has been shown to exhibit a non-linear relationship with regard to its ATPase rate and substrate unfolding rate, especially in more tightly folded substrates (Nager et al., 2011). This is expected since at saturating ATP concentrations ClpX is able to translocate at maximal rates and trap unfolded intermediates, but when the ATPase rate is slowed (by using lower ATP concentrations or by competing with non-hydrolyzable ATPγS) the net translocation rate is also slowed when the unfolded intermediates refold before ClpX can trap them. Thus, at lower ATP hydrolysis rates ATP hydrolysis becomes non-productive and ClpX continually slips on the substrate without productive translocation (Figure 1A). This model for ClpX translocation kinetics has also been supported with single-molecule force experiments (Aubin-Tam et al., 2011; Maillard et al., 2011; Iosefson et al., 2015b; Rodriguez-Aliaga et al., 2016).

In the present study, we ask if the proteasomal ATPases have translocation and unfolding kinetics that are consistent with this model of ClpX, or if its structural and mechanochemical differences allow it to take a different strategy for substrate unfolding. We show that, unlike ClpX, the 19S and PAN proteasomal ATPases resemble a lower velocity, but highly processive motor that is slower than ClpX but does not appear to stall when it approaches the stably folded domain of GFP, but rather it drives through it without slipping. These kinetics are consistent with the hand over hand sequential mechanism of ATP hydrolysis that has been proposed for the proteasomal ATPases (Smith et al., 2011; Kim et al., 2015). These data therefore suggest that proteasomal ATPases, while slower, are more processive and efficient than ClpX and use a different kinetic strategy for unfolding substrates.

## Results

In order to test unfolding ability of PAN, we used the model substrate of GFP with an unstructured ssrA tag fused to its N-terminus (GFPssrA). GFP's fluorescence is dependent on its tertiary structure; therefore, the rate of unfolding can be monitored by following its decrease in fluorescence in real time. As expected, PAN unfolded GFPssrA in an ATP-dependent manner (Figure 1B). The slow loss of GFP fluorescence in the “no ATP” control is attributed to the slow bleaching of GFP with time, which is expected. To determine the catalytic affinity (Km) for GFP we performed a GFPssrA dose response at saturating [ATP] (2 mM). The unfolding rate was determined by calculating the maximum linear rate of the change in GFP fluorescence with time. The Vmax of GFPssrA unfolding was 0.44 ± 0.01 GFPs·PAN^−1^·min^−1^, which indicates that PAN takes ~2 min to unfold a single GFP. This unfolding rate for the proteasomal ATPases is consistent with prior observations (Benaroudj et al., 2003). In addition, the Km was found to be 0.187 μM (Figure 1C). Next, we determined the ATP hydrolysis rate in PAN using a real-time NADH-coupled assay and found the rate of ATP hydrolysis to be 58.5 ± 3.5 ATPs·PAN^−1^·min^−1^ in the absence of substrate and was activated ~1.7-fold to 97.0 ± 2.9 ATPs·PAN^−1^·min^−1^ upon addition of saturating GFPssrA (2 μM), which is also consistent with previous reports (Kim et al., 2015; Figure 1D). The ATP hydrolysis rate we found for PAN is fairly similar to previous reports in the mammalian 26S proteasome, which place the ATPase rates between ~30 and 50 ATPs per minute in the absence of substrate (Hoffman and Rechsteiner, 1996; Kraut et al., 2012), with a ~1.5–2-fold activation upon addition of substrate (Peth et al., 2013). We compared this ATP hydrolysis rate to previously reported ATP hydrolysis rates for the psueudohexameric ClpX. Reported ATPase rates for the ClpX pseudohexamer tend to vary quite a bit (~100–500 ATPs per minute; Martin et al., 2005; Aubin-Tam et al., 2011; Maillard et al., 2011; Nager et al., 2011; Baytshtok et al., 2015; Iosefson et al., 2015a; Rodriguez-Aliaga et al., 2016), but all of these rates are considerably faster than the reported basal rates for the proteasomal ATPases. Addition of substrate to ClpX typically increases its ATP hydrolysis rate, although the degree to which ClpX is activated depends upon the substrate analyzed (Kenniston et al., 2003; Baytshtok et al., 2015; Iosefson et al., 2015a).

**Figure 2:**
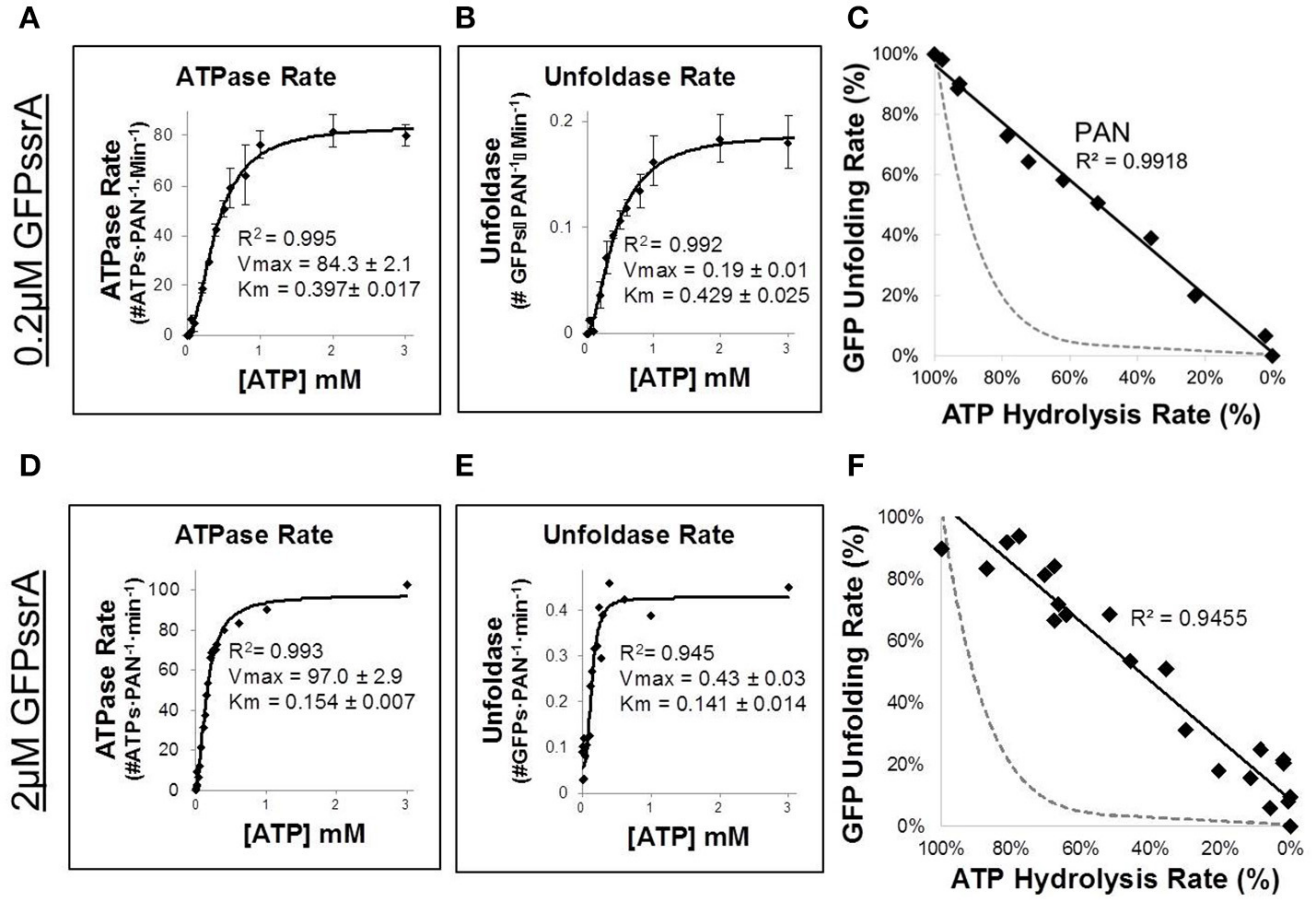
**PAN does not stall when it encounters the unfolded domain of GFP**. **(A,B)** To determine mechanochemical coupling efficiency at ~Km levels of GFPssrA, ATP hydrolysis and GFPssrA unfolding (2 μM) were assessed concurrently, in the same well, using an NADH-coupled ATPase assay combined with GFPssrA unfolding (see Materials and Methods Section). Rate of ATP hydrolysis was measured by loss of NADH absorbance at 340 nm **(A)**, while at the same time GFPssrA unfolding rate was measured by loss of fluorescence at ex/em: 485/510 **(B)**. **(C)** Efficiency of mechanochemical coupling of ATP hydrolysis to GFPssrA was determined by plotting relative percentage ATPase and unfoldase onto a 2 dimensional plot and fitting with a line (*R*^2^ = 0.9918). The dotted gray line is a hypothetical example of an ATPase that stalls (e.g., ClpX), where stalling is defined as <5% of the maximal degradation rate when the ATPase rate is 50% of maximal (Nager et al., 2011). **(D–F)** Same as **(A–C)**, but at saturating GFPssrA substrate concentration (2 μM). Error bars are standard deviations from three independent experiments (*n* = 3).

A longstanding question in the proteasomal ATPase field is how chemical energy from ATP is converted into mechanical work on substrates, and the efficiency of such mechanochemical coupling is informative to mechanism. In ClpX, it was found that at higher ATPase rates, ClpX has quite efficient mechanochemical coupling; however, at lower ATPase rates coupling is less efficient (i.e., at lower ATPase rates, ATP hydrolysis often does not lead to unfolding). This less efficient mechanochemical coupling can be observed by decreasing the rate of ATP hydrolysis by either reducing total [ATP] or competing with non-hydrolyzable nucleotide. In order to test the mechanochemical coupling efficiency of PAN, we simultaneously measured, in real time, the unfolding rate of GFPssrA and PAN's ATPase activity (via absorbance of NADH in a coupled ATPase assay—see Materials and Methods Section). 0.2 μM GFPssrA (~Km) was incubated with PAN at various concentrations of ATP to determine the ATPase (Figure 2A) and unfoldase rates (Figure 2B). To our surprise, Km-values of PAN's ATPase and GFPssrA unfolding matched quite well with one another, with the Km of ATPase activity being 0.397 ± 0.017 μM and the Km for GFPssrA unfolding being 0.429 ± 0.025 μM. This suggested a tight coupling between unfolding and ATPase rates at least around ½ Vmax. We then plotted the GFP unfolding and ATP hydrolysis rates against each other on a single 2-dimensional plot (Figure 2C). Surprisingly, the data was very linear and fit a linear curve with an *R*^2^ of 0.9918. Therefore, PAN exhibits a 1:1 mechanochemical coupling of ATPase and unfoldase activities. In contrast, prior experiments with ATPases that stall (e.g., ClpX) have shown that its ATPase to GFPssrA unfoldase plot is highly non-linear (e.g., when the ATPase rate is ~50%, the unfolding rate drops to <5%). In Figures 2C,F, we show a dotted gray line as an example of what the ATPase vs. unfoldase plot would look like in a stalling ATPase (e.g., ClpX). This non-linear ATPase to GFPssrA unfoldase relationship has been attributed to increased substrate “stalling” and “slipping” upon reaching a globular domain (i.e., GFP's beta-barrel), which results in non-productive ATP hydrolysis (Aubin-Tam et al., 2011; Maillard et al., 2011; Nager et al., 2011; Iosefson et al., 2015b; Rodriguez-Aliaga et al., 2016). Since we found that PAN's ATPase activity is directly proportional (1:1) to GFPssrA unfolding, this data indicates that PAN essentially does not slip when it reaches the folded domain of the GFP beta-barrel. We repeated the experiment using saturating levels of GFPssrA (2 μM) and found that the Km for ATPase activity and GFP unfolding were nearly identical to one another (Figures 2D,E). Consistent with Figure 1C, the Vmax for unfolding was 2-fold higher at saturating [GFPssrA] (0.43 ± 0.03 GFPs·PAN^−1^·min^−1^; Figure 2E) compared to at the Vmax at ~Km concentrations of GFPssrA (0.19 ± 0.01 GFPs·PAN^−1^·min^−1^; Figure 2B). This is expected since the unfolding rate at Km concentrations of GFPssrA should be ½ of the Vmax. Consistent with prior observations, we observed here that saturating levels of GFPssrA stimulated the Vmax for ATPase activity by ~1.7-fold when compared to the no substrate ATPase experiments (Figure 1D), and a ~1.2-fold increase when compared to the 200 nM GFPssrA experiments (Figures 2A,D). Interestingly, we found that in addition to increasing the Vmax, saturating levels of GFPssrA also lowered the Km for ATP hydrolysis and substrate unfolding ~2–3-fold (compare Km-values in Figures 2A,B to Km-values in Figures 2D,E). This may suggest an underlying mechanism for substrate stimulated ATPase activity, which is well-established in the literature. In addition, the similar Km between ATPase and unfoldase activities at saturating substrate levels is consistent with the linear fit (*R*^2^ = 0.9455) that we observe when plotting ATP hydrolysis against GFP unfolding (Figure 2F), similar to Figure 2C. Thus, even when all PAN complexes are bound to a GFPssrA the rate of ATP hydrolysis is tightly coupled to GFP unfolding. In other words, hydrolysis of ATP by PAN almost always results in a successful translocation event, even when it meets a globular domain.

**Figure 3:**
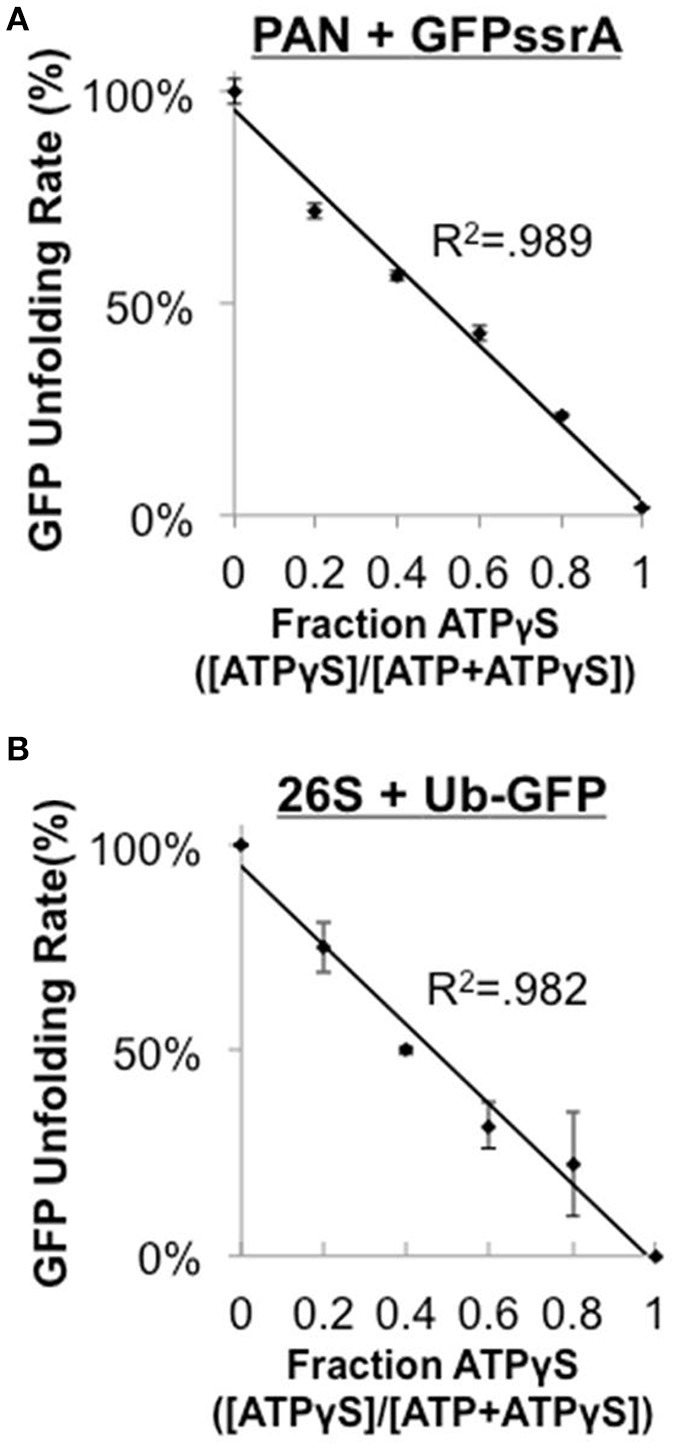
**The eukaryotic 26S does not stall when it encounters the folded domain of GFP. (A)** PAN's ATPase rate was slowed by competing with increasing ratios of ATPγS:ATP (2 mM total nucleotide), and GFPssrA (0.2 μM) unfolding rate was assessed as in Figure 1A. Data fit a line with an *R*^2^ = 0.989. **(B)** 25 nM of purified rabbit 26S was incubated with 100 nM Ub^4^(lin)-GFP^35^ and was analyzed as in **(A)**. Data fit a line with an *R*^2^-value of 0.982. “Stalling” is defined in Figure 2. Error bars are standard deviations from three independent experiments (*n* = 3).

The eukaryotic 19S ATPases are homologous to PAN, however, the 19S forms a heterohexameric ring and has many additional associated non-ATPase subunits while PAN forms a homohexameric ring and has no known non-ATPase subunits. Therefore, it was unclear whether the 1:1 mechanochemical coupling of ATPase rate to substrate unfolding that we observed in PAN would be a general property of proteasomal ATPases, or whether it would only apply to the archaeal proteasomal ATPases. Therefore, we sought to determine whether the eukaryotic 26S (i.e., 19S–20S complex) also had a similar linear relationship between its ATPase and unfoldase activity. The Matouschek group generously provided us with a novel 26S substrate, Ub^4^(lin)-GFP^35^-His^6^, suitable for use with *in vitro* 26S unfolding assays. Such a substrate is very useful for mechanistic studies since it allows for the analysis of ubiquitin- and ATP-dependent degradation using the purified 26S proteasome. For the 26S proteasome to remain functional it requires the persistent presence of ATP, so we could not assess coupling of ATPase and substrate unfolding using the ATP dose response as was done in Figure 2 for PAN because low ATP concentrations would induce disassembly of the 26S proteasome (Thompson et al., 2009). Instead, we slowed ATPase rate by competing ATP with the largely non-hydrolyzable ATP analog, ATPγS (which by itself stabilizes the 26S complex as does ATP). We first performed this ATPγS competition experiment in PAN and found that as the ATPγS:ATP ratio increased, GFPssrA unfolding rate decreased in a 1:1 linear relationship with the ATPγS:ATP ratio (*R*^2^ = 0.989; Figure 3A). This is consistent with and further supports our observations with the ATP dose response method in Figures 2C,F, and it demonstrates that the ATPγS:ATP ratio method mimics a linear decrease in ATP hydrolysis activity in PAN similar to the ATP dose response. We next performed a similar ATPγS competition experiment using the Ub^4^(lin)-GFP^35^ substrate and the eukaryotic 26S proteasome and were surprised to find that the 26S had similar 1:1 unfolding kinetics to that observed in PAN (Figure 3B) with a strong linear fit (*R*^2^ = 0.982). These ATPγS competition experiments demonstrate that ATP hydrolysis and unfolding are also tightly coupled in ubiquitin-dependent protein degradation by the eukaryotic 26S proteasome. In addition, this indicates that the tight mechanochemical coupling between ATP hydrolysis and unfolding ability is shared between PAN and the 26S and thus it is expected to be a general property of the proteasomal ATPases despite their structural differences.

## Discussion

**Figure 4:**
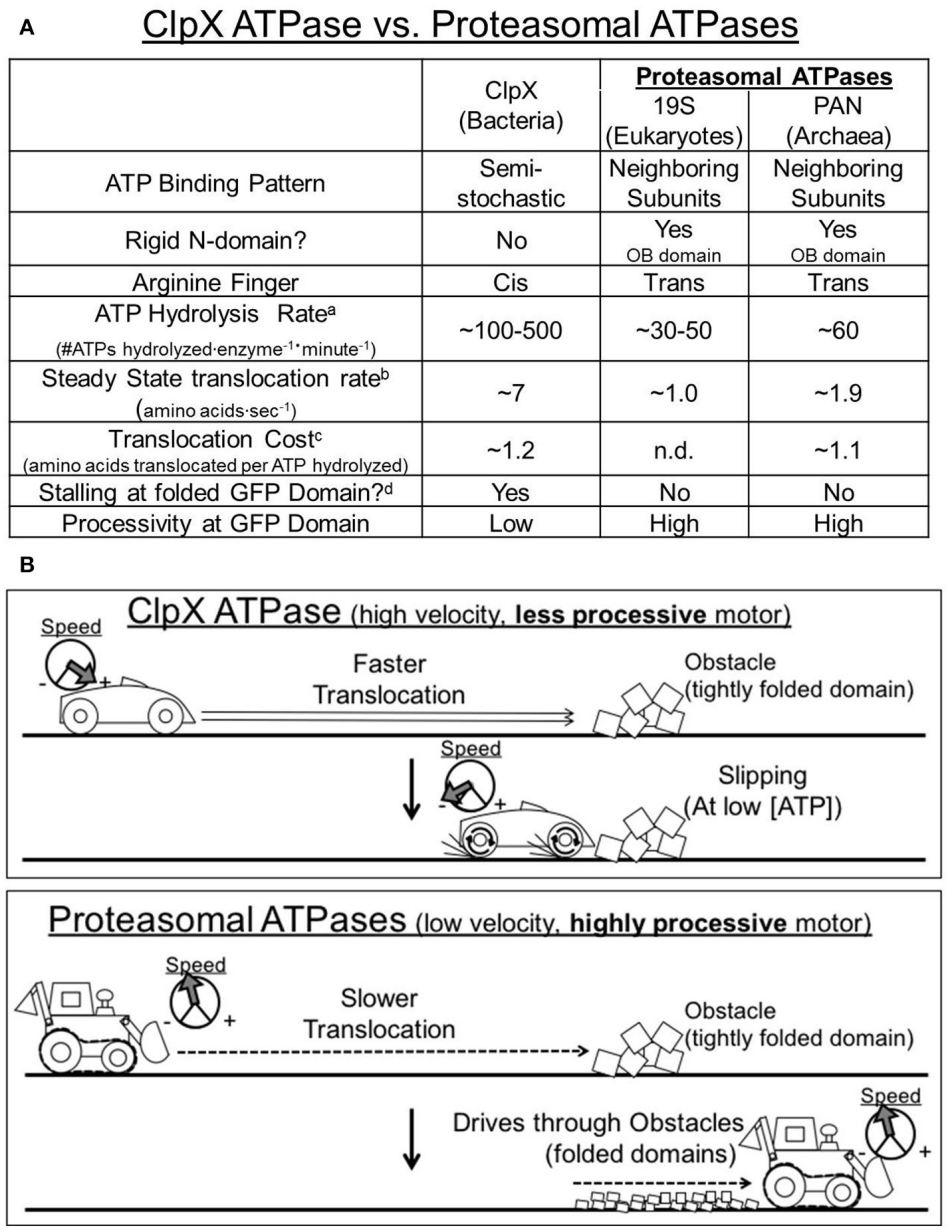
**Comparison of the unfolding kinetics for the Proteasomal ATPases vs. ClpX. (A)** Summary of ClpX and the proteasomal ATPases' unfolding kinetics taken from experiments performed in this manuscript as well as by other groups (cited in main text). Footnotes: ^a^ATP hydrolysis rate in the absence of substrate. ^b^Steady state translocation rates are taken from mean unfolding rates with non-stalling substrates. ^c^Translocation cost is calculated as the rate of steady state translocation on a non-stalling substrate, divided by the ATPase rate of the enzyme on that same substrate. ^d^Stalling is defined as <5% of max unfolding rate at 50% max ATPase activity (Nager et al., 2011). **(B)** Working model: ClpX ATPases resemble a higher velocity, less processive motor that is prone to slipping. ClpX translocates rather quickly along a loosely folded protein domain. However, at low ATP concentrations, ClpX is unable to drive through tightly folded protein domains, and thus undergoes multiple slips and stalls, and can even dissociate from the protein completely. Proteasomal ATPases resemble a lower velocity, more processive motor. The proteasomal ATPases translocate more slowly along a loosely folded protein domain, but even at these lower speeds the proteasomal ATPase is able to drive through more tightly folded domains (i.e., GFP) without significant slipping or stalling.

Previous studies reveal that the bacterial ClpX pseudohexamer resembles a higher velocity motor. It also has a correspondingly quick steady-state translocation rate: for example ~7 amino acids per second on the “non-stalling” substrate, cp6^SF^GFPssrA (Nager et al., 2011). However, when ClpX reaches a tightly folded domain “stalling” and “slipping” can occur, whereby it loses its grip on the substrate and the substrate is often released, resulting in unproductive ATP hydrolysis (Aubin-Tam et al., 2011; Maillard et al., 2011; Nager et al., 2011; Iosefson et al., 2015b; Rodriguez-Aliaga et al., 2016; Figures 4A,B). In contrast, the proteasomal ATPases hydrolyze ATP considerably more slowly than does ClpX and we estimate that proteasomal ATPases translocate on non-stalling substrates at an average rate of ~1.0–1.9 amino acids per second, or about ~3–7 times more slowly than ClpX. Interestingly, despite these differences in translocation velocity both PAN and ClpX show a similar cost for non-stalling translocation at a mean of ~1.1–1.2 amino acids translocated per ATP that is hydrolyzed (Figure 4A). Despite this similarity, here we find for the proteasomal ATPases that even at low ATPase rates ATP hydrolysis is tightly coupled with translocation, which is the force that drives unfolding. This is consistent with a lack of substrate “slipping,” and indicates that proteasomal ATPases are more efficient and processive than ClpX particularly when they reach a folded domain. Therefore, the proteasomal ATPases operate at a lower velocity, but also have higher processivity since they do not slip or lose grip on the substrate (Figures 4A,B). This suggests that ClpX and PAN utilize different kinetic strategies to unfold proteins: ClpX uses a fast translocation strategy to trap unfolded intermediates, while the proteasomal ATPases use a slower but more processive and efficient kinetic strategy to drive through unfolded domains with a tight mechanochemical coupling between ATP hydrolysis and translocation events.

What functional characteristics in these ATPases could cause these different kinetic strategies for unfolding proteins? One possibility is the sequential vs. semi-stochastic mechanisms that have been proposed for the proteasomal ATPases vs. ClpX (Figure 4A). It could be expected that a semi-stochastic ATP-hydrolysis mechanism could lead to states of the ring where all ATPs are hydrolyzed, leaving ClpX in an ADP-bound state only. Since ATP binding drives substrate association, this could lead to loss of substrate affinity and slipping, especially when ATP is limiting. In contrast, it has been proposed that the proteasomal ATPases use a sequential single subunit progression mechanism for ATP hydrolysis (Kim et al., 2015). In this model, at least one ATPase subunit is always bound to an ATP, supporting constant affinity for the substrate, which would be expected to prevent slipping. In this model it would thus be expected that most hydrolysis events are coupled to translocation events, which is supported by our data presented here. This tight mechanochemical coupling can be explained by two different models for the proteasomal ATPases: (1) ATP hydrolysis has sufficient power to forcibly unfold GFP with each power stroke, allowing the ATPase to drive through unfolded domains or (2) ATP hydrolysis does not occur in any one subunit until translocation can take place. These two models could represent differences in the “power stroke” vs. “Brownian ratchet” mechanisms, and many ATPase motors exhibit a blending of both of these mechanisms, but neither of these have been determined for the proteasomal ATPases. However, both models are consistent with the data we have shown here. It's also possible that other structural differences between ClpX and the proteasomal ATPases could play a role in the unfolding kinetics. For example, the proteasomal ATPases have trans-arginine fingers (vs. cis-arginine fingers in ClpX), which constitutes an arginine that allows one subunit to contact the gamma phosphate of the ATP bound to the Walker A/B sites in its neighboring subunit. This arginine is critical for the effects of ATP-binding in the proteasomal ATPases, which include promoting substrate binding, and the association of PAN/19S with the 20S core particle and gate-opening. The placement and allosteric role of this trans-arginine is a fundamental difference between the proteasomal ATPases and ClpX. In addition, the role of the trans-arginine finger combined with the single subunit progression model produces a hand-over-hand translocation model that would be expected to exhibit a high “grip” strength mechanism that allows for high substrate binding affinity even at low ATP (Kim et al., 2015). The proteasomal ATPases also contain a rigid ring of OB domains that substrates are threaded through during translocation. This threading ring generates a rigid platform that folded domains can be pulled against during translocation to cause unfolding. The lack of such a domain in ClpX means that globular domains are pulled into and against the ATPase domains themselves during translocation (especially for the ΔN-ClpX which is used in most of the *in vitro* experiments that study translocation), which could sterically alter their activity during forceful pulling, and could perhaps cause slipping as well (Figure 4A).

So why might these two distinct mechanisms have evolved for unfolding proteins? In bacteria, ssrA tags are added to the C-terminus of translationally stalled proteins on ribosomes. In fact, ~1 in 200 translated proteins are tagged by ssrA, and of these, >90% are degraded by ClpX(P) (Lies and Maurizi, 2008). The vast majority of these translationally stalled proteins will produce truncated proteins, which will typically prevent proper folding, thus destabilizing these proteins. These truncated proteins must also be rapidly degraded in order to prevent aggregation and/or toxicity to the cell. Therefore, a high-velocity unfoldase like ClpX is well-suited to quickly handle such proteins, and perhaps ClpX would only rarely be expected to encounter a more tightly folded protein, which could be handled by other ATPases in bacteria such as ClpA. On the other hand, here we have observed that the proteasomal ATPases resemble a lower velocity motor with a more processive and efficient translocation mechanism. Why might this be? The proteasome degrades most proteins in the cell, both unfolded as well as fully folded, functional proteins. Thus, in order for the proteasome to function optimally for this job it must be able to routinely handle more tightly folded domains than ClpX typically encounters. The high processivity, low velocity characteristics that we have observed here for the proteasomal ATPases seem to be optimized for its specific client pool of proteins that demand reliable degradation of folded and functional proteins. Therefore, we propose that the need to unfold and degrade most folded proteins in the cell is the reason that the proteasomal ATPases use a slower but more processive strategy for protein unfolding and degradation.

## Materials and methods

### Materials, plasmids, and protein purification

PAN, GFPssrA, and T20S were prepared as described (Smith et al., 2005, 2007). The purest available forms of ATP, and ATPγS were purchased from Sigma and stored at −80°C until use. Rabbit muscle 26S proteasome was purified by the previously described UBL-UIM method (Besche et al., 2009) and were exchanged with reaction buffer by rapid spin column or by dialysis (4 h) immediately prior to use.

Ub^4^(lin)-GFP^35^-His^6^ plasmid was a generous gift from Andreas Matouschek and his lab. Plasmids were transfected into DH5α cells, and 1L cultures were grown at 37° at 300 RPM shaking, and induced with IPTG at OD_600_ = 0.8 for 4 h. Cell pellets were resuspended in Buffer A (50 mM Tris pH 7.5, 5% glycerol, 300 mM NaCl, 20 mM Imidazole) with 1X protease inhibitor cocktail. Cells were lysed via sonication and spun at 20000 × g for 30 min. Supernatant was loaded onto Nickel-NTA, washed with 10 CV Buffer A, and eluted with Buffer B (Buffer A w/ 300 mM Imidazole). Fractions containing Ub^4^(lin)-GFP^35^-His^6^ were pooled based on fluorescence (ex/em: 485/510) and SDS-PAGE. Pooled fractions were concentrated and further purified using size-exclusion chromatography (GE Superose 12 column). Purest fractions were exchanged into 50 mM Tris pH 7.5 + 5% glycerol.

### ATPase and GFPssrA unfolding assays

ATP hydrolysis was measured by reading the loss of NADH absorbance at 340 nm in an NADH-coupled ATP regenerating system (50 mM Tris pH 7.5, 5% glycerol, 20 mM MgCl2, 2 U/μl Pyrivate Kinase, 2 U/μl Lactate dehydrogenase, 3 mM phosphoenolpyruvate, and 0.2 mg/ml NADH, and indicated [ATP]). GFPssrA unfolding was assessed by loss of fluorescence at ex/em: 485/510. For the unfolding experiments, reaction buffer (50 mM Tris pH 7.5, 5% glycerol, 20 mM MgCl2) was incubated with 50 nM PAN, 400 nM T20S, and 0.2 nM GFPssrA (or 25 nM 26S and 100 nM Ub^4^(lin)-GFP^35^-His^6^ for experiments with 26S) and 2 mM ATP (or with indicated ATPγS:ATP ratios with 2 mM total nucleotide). GFP fluorescence loss (ex/em: 485/510) was measured every 20 s in a Biotek 96 well-plate reader to obtain unfolding rates. Error bars represent standard deviations from at least three independent experiments (*n* ≥ 3).

ATP hydrolysis and GFPssrA unfolding were assessed concurrently in a Biotek 96 well-plate reader by measuring NADH absorbance loss alongside GFPssrA fluorescence loss. The ATP regenerating system buffer (above) was incubated with indicated [ATP] (0–3 mM), 50 nM PAN, 400 nM T20S, and 0.2 μM or 2 μM GFPssrA. Rates of ATP hydrolysis and GFPssrA unfolding were extrapolated and Vmax and Km-values were obtained by non-linear regression analysis on Sigmaplot using the Hill equation. Error bars are standard deviations from at least three independent experiments (*n* ≥ 3).

## Author contributions

AS purified most proteins used in the manuscript, RA purified the Ub-GFP substrate. AS designed, performed, and analyzed the various experiments in this manuscript with input from RA and DS. Manuscript preparation was done by AS and DS. All authors reviewed the results and approved the final version of this manuscript.

## Funding

This work was supported by NIH-R01GM107129 to DS and by F31GM115171 to AS.

### Conflict of interest statement

The authors declare that the research was conducted in the absence of any commercial or financial relationships that could be construed as a potential conflict of interest.
